# Semantics Count in the Description of the Interactions Between Bacteria and Bacteriophage

**DOI:** 10.1089/phage.2024.0063

**Published:** 2025-03-17

**Authors:** Brandon A. Berryhill, Bruce R. Levin

**Affiliations:** ^1^ Department of Biology, Emory University, Atlanta, Georgia, USA.; ^2^ Program in Microbiology and Molecular Genetics, Graduate Division of Biological and Biomedical Sciences, Laney Graduate School, Emory University, Atlanta, Georgia, USA.

Bacteriophage (phage) have played a major role in the development of molecular biology as we know it.^1,2^ Phage continue to be used as a tool for understanding basic biology, see for example the recent Nobel Prize for Phage Display.^3^ However, as a consequence of the increased frequency of infections with antibiotic-resistant bacteria and subsequent increase in treatment failure, there has been a renewed interest in using bacteriophage to treat and prevent bacterial infections.^3,4^ Much is known about the interactions of bacteria and their phages, including the various defense mechanisms bacteria employ to survive phage infection.^5,6^ It has struck the authors in the last 2 years that there is a major ambiguity in the field, which, at first, may seem purely semantic but needs to be corrected as it is fundamental to understanding the ecology and evolution of these viruses and their host. That is, the difference between what are commonly called resistance and immunity.

We believe these two defenses bacteria employ to prevent succumbing to phage infection are substantially different and should be considered separately. For resistance, the phage does not adsorb to the bacteria and is not lost from the population. In contrast, for immunity, the phage does adsorb to the bacteria, inject their DNA, and thus are removed from the free virus population. Stated another way, for a bacterium to be resistant to a phage it must be refractory to that virus.

There are many different molecular mechanisms that underlie both resistance and immunity. Common mechanisms of resistance include receptor site mutations and the display of sterically inhibiting antigens.^7–10^ There is an abundance of immunity mechanisms including restriction-modification, abortive infection, and most CRISPR systems.^11–14^ However, as with all things in biology, some elements blur the line between these two defenses. The best examples are temperate phages, which convey superinfection immunity, that is, the inability to be infected with the same bacteriophage twice.^15^ Superinfection immunity can work at many different stages of the phage lifecycle such that the bacteria carrying the prophage of the virus, lysogens, can appear either resistant or immune to the corresponding phage. In the classical case of the phage lambda (λ), when the λ genome is integrated into that of the host bacteria as a prophage, free λ phage can adsorb but are prevented from replicating.^16^ These bacteria would not appear refractory. However, some temperate phages, such as those of Pseudomonas, code for genes that, when expressed inside of the bacterial host, lead to changes in the phage receptor, preventing the adsorption of the same phage.^15^ These bacteria would appear refractory. Despite the heterogeneity in mechanism, we argue that the distinction and the test between resistance and immunity is unambiguous. If phage adsorb and are lost from the environment, then the bacteria is immune. If the phage do not adsorb, then the bacteria appear refractory and thus would be resistant. This test is critical to perform when concluding whether a bacterium is immune or resistant, as in recent studies, we have found that phage-resistance bacteria dominate in natural communities where we would intuitively believe immunity should be the dominant character.^9,17^

This distinction is not purely semantic. There are population and evolutionary consequences to this distinction. The most apparent of which is the ability of immune cells to remove phage from the environment and thereby protect coexisting bacteria, which are sensitive to the phage.^18,19^ Secondarily, there are major differences in the impact of each to the target bacterium. With immunity mechanisms, the bacterial membrane is disrupted, even if only minorly so. This can be seen in that bacteria that are immune to a phage still undergo the SOS stress response when infected with the phage for which they are immune.^17^ This is not the case for bacteria that are resistant to phage. Both of these factors are critical when discussing the maintenance of phage in their natural habits. Recently, there has been an incredible amount of interest in what phage are doing to shape the species and strain dynamics of the enteric microbiome of humans and what implications these viruses have in human health.^20–22^ This is particularly critical in environments where temperate phages abound, which appears to be the case in the enteric microbiome.^20^ These questions cannot be addressed without considering whether the underlying bacterial populations are immune or refractory to the coexisting phage populations.

There are many recent studies describing the various mechanisms by which bacteria avoid succumbing to phage infection, especially in the context of molecular biology and bioengineering.^23,24^ However, we argue many of these systems are immunity rather than resistance. Indeed, many of the now famous tools of molecular biology that have emerged from the study of phages, such as restriction endonucleases, CRISPR-Cas, and Retrons, are mechanisms of immunity.

Moving forward, we recommend when characterizing phage defense systems, that the distinction be made as to whether or not the bacteria are refractory to the infecting virus. This distinction is particularly salient when performing retrospective analyses on populations that have already reached some sort of steady state such as established communities. Moreover, we specifically recommend describing the bacteria to which the phage do not adsorb as refractory rather than resistant as a way to reduce the ambiguity of these defense mechanisms.
